# Blood nulling versus tissue suppression: Enhancing integrated VASO and perfusion (VAPER) contrast for laminar fMRI

**DOI:** 10.1162/imag_a_00453

**Published:** 2025-01-21

**Authors:** Yuhui Chai, Linqing Li, Rüdiger Stirnberg, Laurentius Huber, Tony Stöcker, Peter A. Bandettini, Bradley P. Sutton

**Affiliations:** Beckman Institute for Advanced Science and Technology, University of Illinois at Urbana-Champaign, Urbana, IL, United States; Functional MRI Core, National Institute of Mental Health, National Institutes of Health, Bethesda, MD, United States; German Center for Neurodegenerative Diseases, Bonn, Germany; Department of Physics and Astronomy, University of Bonn, Bonn, Germany

**Keywords:** laminar fMRI, cortical layer, cortical depth, high-resolution fMRI, magnetization transfer

## Abstract

Cerebral blood volume (CBV) and cerebral blood flow (CBF)-based functional magnetic resonance imaging (fMRI) have proven to be more laminar-specific than blood-oxygen-level-dependent (BOLD) contrast fMRI, but they suffer from relatively low sensitivity. In previous work, we integrated CBV and CBF into one contrast using DANTE (Delay Alternating with Nutation for Tailored Excitation) pulse trains combined with 3D echo-planar imaging (EPI) to create an integrated blood volume and perfusion (VAPER)-weighted contrast (Chai et al., 2020). Building on this, we have now introduced a magnetization transfer approach to induce a tissue-suppression-based VASO (vascular space occupancy) effect and incorporated it with the VAPER technique to boost the overall sensitivity while maintaining superior laminar specificity, all without altering the original VAPER sequence timing scheme. This magnetization transfer (MT)–VAPER fMRI acquisition alternates between DANTE blood-nulling and MT-tissue-suppression conditions, generating an integrated VASO and perfusion contrast enhanced by MT. Both theoretical and experimental evaluation demonstrated an approximately 30% enhancement in VAPER sensitivity with MT application. This novel MT–VAPER method was empirically validated in human primary motor and visual cortices, demonstrating its superior laminar specificity and robust reproducibility, establishing it as valuable non-BOLD tool for laminar fMRI in human brain function research.

## 1 Introduction

Functional magnetic resonance imaging (fMRI), since its development (Bandettini et al., 1992;Belliveau et al., 1991;Kwong et al., 1992;Ogawa et al., 1990,^1992^), has become an indispensable tool in human cognitive neuroscience, enabling non-invasive studies of brain function. The advent of ultra-high-field (≥7T) human MRI scanners has significantly advanced the spatial resolution of fMRI, enabling sub-millimeter distinctions in cortical depth (Koopmans et al., 2010;Zimmermann et al., 2011). Although these depths do not map directly onto cytoarchitectonic cortical layers—molecular (I), external granular (II), external pyramidal (III), internal granular (IV), internal pyramidal (V), and multiform (VI) (Fatterpekar et al., 2002)—they provide detailed contours that broadly correspond to functional layers: superficial depths with supragranular layers (I to III), middle depths with the granular layer (IV), and deep depths with infragranular layers (V and VI) (Waehnert et al., 2014). Cortical layers are recognized for their distinct functional roles (Lamme et al., 1998;Self et al., 2019). Both layer-specific activity (Lawrence et al., 2017;Muckli et al., 2015) and connectivity (Chai et al., 2024;Huber, Finn, et al., 2021;Rockland, 2019) can be used to dissociate bottom-up and top-down cognitive processes and to define functional hierarchy among cortical areas (Stephan et al., 2017). Mapping these activities and connections across cortical layers is, therefore, crucial for advancing our understanding of human cognition.

Despite the great potential of laminar fMRI research in humans, its broader application faces technical challenges. The gradient echo (GE) blood-oxygenation-level-dependent (BOLD) contrast is the most prevalent technique for human functional brain mapping (Ogawa et al., 1990) due to its high sensitivity among non-invasive fMRI methods. However, the GE–BOLD signal is prone to the draining vein effect, and thus its layer-specific microvasculature signal is often washed out by dominant signal from ascending and pial veins (S. G. Kim & Ogawa, 2012). Selective sensitivity of cerebral blood volume (CBV) and flow (CBF) has been demonstrated to provide improved spatial specificity to the activated parenchyma and better localization of layer-specific activity in animal models (T. Jin & Kim, 2008a). Thus, CBV and CBF-based fMRI measurements, such as ASL (arterial spin labeling) (Kashyap et al., 2021;Shao et al., 2021), VAPER (integrated blood volume and perfusion) (Chai et al., 2020), and VASO (vascular space occupancy) (Huber et al., 2014;Lu et al., 2005) imaging, have been explored and demonstrated to be more layer-specific than traditional BOLD imaging.

Although CBV and CBF-based fMRI provide superior laminar specificity, they are constrained by relatively low sensitivity. For example, the VAPER method shows a sensitivity reduction of more than half when BOLD contributions are excluded (Chai et al., 2020). Similarly, VASO inherently has a low signal-to-noise ratio (SNR), with only 10–20% of tissue signal remaining at the time of blood nulling (Hua et al., 2009). It has been demonstrated that integrating a magnetization transfer (MT) technique with VASO can significantly improve its contrast-to-noise ratio (CNR) by around 40% (Hua et al., 2009). By incorporating MT presaturation pulses, the tissue signal recovers more rapidly, leading to a stronger signal at the same blood-nulling time, thereby enhancing the VASO signal and contrast-to-noise ratio (Hua et al., 2009).

In this study, we applied MT to induce a tissue-suppression-based VASO effect and integrated it with the VAPER technique to boost overall sensitivity while maintaining superior laminar specificity over BOLD (both GE–BOLD and MT–BOLD), all without extending the repetition time of the original VAPER sequence. The original VAPER sequence was designed to acquire fMRI images alternating between DANTE blood nulling for VASO contrast and control condition for perfusion weighting post-DANTE (Chai et al., 2020). Here we modified the sequence by replacing the control condition with MT tissue suppression to achieve further CBV weighting. Diverging from the traditional MT pulses such as single off-resonance or short length binomial RF pulses, our approach employs a long stream of small flip angle binomial pulses, which allows for a flexible combination with echo-planar imaging (EPI) acquisitions of variable segmentation, complying with the specific absorption rate (SAR) requirement at human 7T scanners (Chai, Li, et al., 2021). In the following sections, we detail the contrast mechanisms, sequence design, and demonstrate its application in laminar fMRI research.

## 2 Theory

The general scheme for generating MT-enhanced VAPER (MT–VAPER) contrast involves alternating fMRI image acquisitions between two specific conditions: DANTE blood nulling and MT tissue suppression. In the DANTE phase, the blood signal within the human brain’s vasculature is nearly nulled to achieve a VASO contrast. Following this, in the MT condition post-DANTE, fresh blood from outside the coil coverage flows into the imaging area, replacing the nulled blood and generating a perfusion-weighted contrast, while at the same time, the tissue signal is suppressed to create a contrast opposite to the blood-nulling VASO. The signal manipulation between DANTE blood-nulling and MT-tissue-suppression conditions can produce an integrated VASO and perfusion contrast enhanced by MT (MT–VAPER), detailed further in subsequent sections.

### 2.1 Blood signal nulling by DANTE

**Fig. 1.**
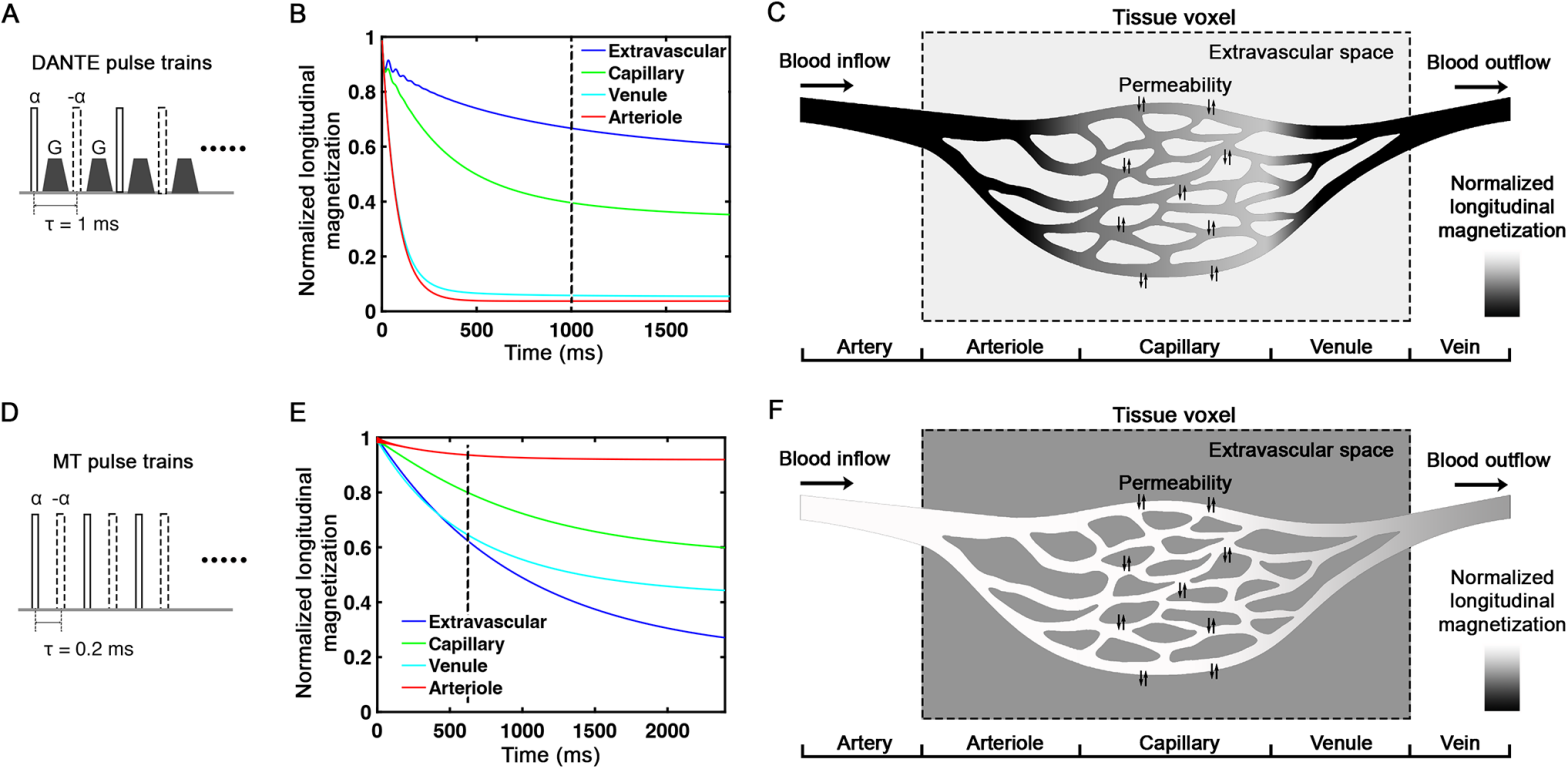
The effect of DANTE and MT pulse trains on the signal of vascular and tissue compartments. (A) DANTE pulse trains consist of a series of low flip angle (α, around 9 degree) RF pulses with alternating polarity (solid rectangle for α, dashed rectangle for -α), interspersed with gradient pulses. (B) The longitudinal magnetization evolution of each compartment as a function of the time length of DANTE pulse trains. The dashed line marks the approximate length of pulses typically applied during a volume TR in fMRI. (C) DANTE suppression effects illustrated in the four-compartment model (dark areas represent more signal attenuation). The blood signal in arteries, arterioles, venules, and veins (flowing faster than 2 mm/s) is mostly suppressed by DANTE, while capillary blood signal is only partially attenuated due to inflow from arterioles. Different vascular compartments are divided along the horizontal axis as marked in the bottom. The tissue voxel’s remaining signal is mainly contributed by the extravascular compartment. (D) MT binomial pulse train consists of a series of low flip angle (α, around 11 degree) pulses with alternating polarity. (E) The longitudinal magnetization behavior of each compartment as a function of the time length of MT pulse trains. The dashed line marks the approximate number of pulses we applied during a volume TR in fMRI due to the SAR limit of the scanner. (F) MT suppression effects illustrated in the four-compartment model, with darkness indicating signal suppression. Extravascular tissue signals undergo relatively greater suppression, while the signal in arteriole blood is predominantly retained. The intensity of the blood signal in capillaries is in intermediate due to the permeability exchange between tissue and capillary.

DANTE pulse trains (Fig. 1A) are designed to selectively attenuate signals from moving spins exceeding a certain velocity cutoff (2 mm/s) while largely preserving stationary tissue signals (Li et al., 2012). As it is applied in a non-selective hard pulse manner, DANTE establishes a flow-crushing effect throughout the RF-transmit field. Using a head-only RF-transmit coil at 7T, this effect is limited to the blood signals within the human head, leaving external signals unaffected.

In the human brain, the blood in arteries, arterioles, venules, and veins typically travels faster than the DANTE cutoff velocity (Liu et al., 2017) and thus, their signal can be almost fully attenuated by the DANTE pulse train. In contrast, the blood velocity in capillaries is generally below 1 mm/s (Liu et al., 2017), and, therefore, its signal can survive from the direct DANTE suppression. However, since arteriole blood (signal nearly fully suppressed by DANTE) continuously flows into capillaries, this influx dilutes the original capillary blood, thus diminishing its signal.

This DANTE suppression effect is incorporated into a four-compartment model, which is schematically depicted inFigure 1B and C. We define a gray matter (GM) parenchyma voxel consisting of tissue and three microvascular compartments (arterioles, capillaries, and venules, up to about 200 μm in size) (Donahue et al., 2006). For detailed physiological parameters of each compartment, refer toChai et al. (2020). The evolution of longitudinal magnetization within each compartment during DANTE pulse trains is numerically simulated (equations inAppendix), employing a flip angle (α) of9°, pulse duration of 90 µs, and a pulse interval (τ) of 1 ms. With more DANTE pulses applied, the longitudinal magnetization of arterioles and venules decays rapidly, stabilizing at a minimal level after 200–300 pulses. Since venules keep receiving the partially suppressed blood from capillaries, their signal is slightly less suppressed than that of the arterioles. Extravascular magnetization is largely preserved compared with the intravascular blood signal. The evolution of capillary magnetization falls intermediate between that of arteriole/venule and extravascular tissue.

### 2.2 Tissue signal suppression by MT

MT pulse train (Fig. 1D) can be used to suppress tissue signal while its effect on blood is negligible. This is achieved using a binomial pulse train (Chai, Li, et al., 2021) that selectively saturates macromolecular protons (MP) within tissue, which, upon magnetization transfer to water hydrogen protons (WP), induces tissue signal suppression.

This approach utilizes a long stream of small flip angle (α, around 11 degrees) binomial RF pulses (Fig. 1D), facilitating a flexible combination with EPI acquisition of large brain coverage while complying with the SAR limit of a 7T human scanner. Although each binomial pulse pair only slightly saturates the macromolecular protons, a cumulative saturation and transfer effect is achieved as the spin-lattice relaxation (0.4 to 2 s^-1^), and two-pools exchange rate (1.45 s^-1^) (Wang et al., 2020) are much slower than the binomial pulse rate (5000 pulses/s in MT preparation).

**Fig. 2.**
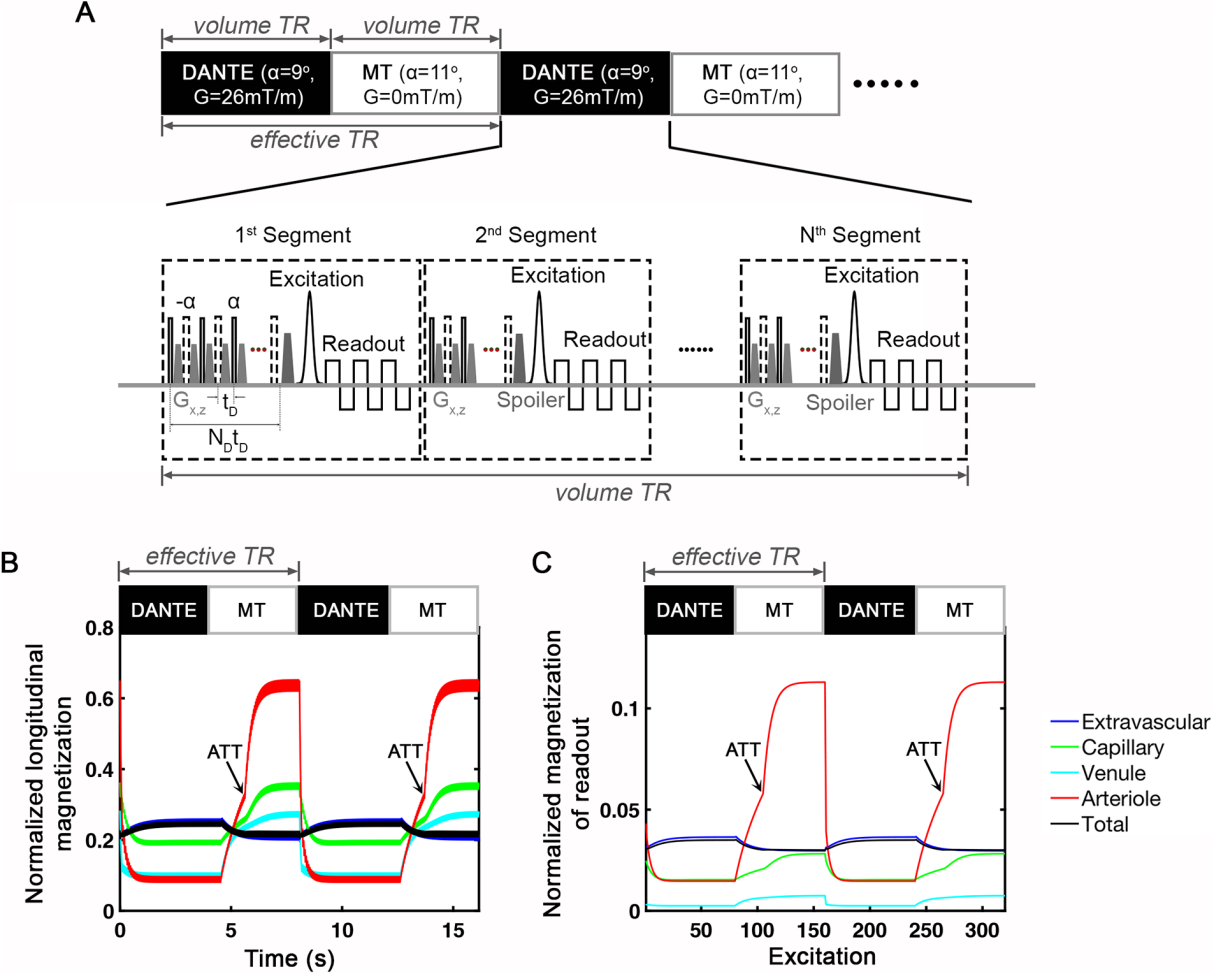
Sequence design for VAPER/MT 3D-EPI and the magnetization of each compartment as a function of time and excitations. (A) During the DANTE blood-nulling condition, DANTE pulse trains are applied before each readout segment of a 3D-EPI acquisition. In the MT-prepared image acquisition, the sequence design is the same as the DANTE condition, except that DANTE gradient pulses are switched off and the RF power of binomial pulse train is maximized. In fMRI data acquisition, interleaved MT and DANTE volumes are acquired. (B) and (C) depict the expected magnetization evolution of each vascular and tissue compartment in longitudinal and readout direction, respectively. ATT represents the arterial transit time. After ATT, fresh blood signal flows into the voxel microvasculature and this boosts the signal of all compartments, especially signal in arterioles. Along the x-axis of time and excitation, the black–white blocks at the top of (B) and (C) mark the time interval of consecutive DANTE and MT acquisitions. Curves in different colors refer to different compartments. The black curves refer to the total signal of a voxel. The y-axis is normalized to the equilibrium magnetization of the respective compartments.

This cumulative suppression mechanism is modeled within the same four-compartment framework (Fig. 2E, F). The evolution of longitudinal magnetization of each compartment during MT pulse train is numerically simulated (equations inAppendix) with a flip angle (α) of11°, pulse duration of 90 µs, and pulse interval (τ) of 0.2 ms. With more MT pulses applied, the longitudinal magnetization of extravascular tissue undergoes relatively greater suppression, whereas that in arteriole blood is predominantly retained. Capillary magnetization is moderately impacted, reflecting a permeability-driven exchange with surrounding tissue. Magnetization in venules declines more rapidly than other vascular compartments, owing to their lower T_2_value, which signal is negligible in readout due to the further fastest T_2_* decay.

### 2.3 Sequence design

Our fMRI pulse sequence is designed to alternate acquisitions between two states: DANTE blood nulling and MT tissue suppression. In the DANTE state, DANTE preparation modules are applied before each segment of a multi-shot 3D-EPI acquisition (Poser et al., 2010) with skipped-CAIPI sampling (Stirnberg & Stöcker, 2021) (Fig. 2A). For the 1^st^segment, around 100 DANTE pulses are applied to enhance the initial blood signal attenuation, followed by 20–40 DANTE pulses in subsequent segments (2^nd^to N^th^) to maintain a consistently low blood signal. Similar to the VASO technique (Lu et al., 2003), the image signal from the DANTE state is sensitive to CBV changes. In the MT state, the sequence arrangement is the same as that in DANTE state except that DANTE gradient pulses are switched off and the RF power of the binominal pulses are maximized. Owing to the head-only RF-transmit coil at 7T, the blood outside of the coil coverage is not affected by previous DANTE suppression and will flow into the image volume to replace the nulled blood during MT state, which generates a perfusion-weighted signal. At the same time, tissue signal is suppressed by MT to induce an effect opposite to the lood-nulling VASO. The MT image signal is thus sensitive to both CBF and CBV changes. The interleaved acquisition of DANTE and MT 3D-EPI sequence enables a controlled alternation of blood and tissue signals across fMRI time series, as simulated inFigure 2B and C.

### 2.4 Generation of MT–VAPER contrasts

**Fig. 3.**
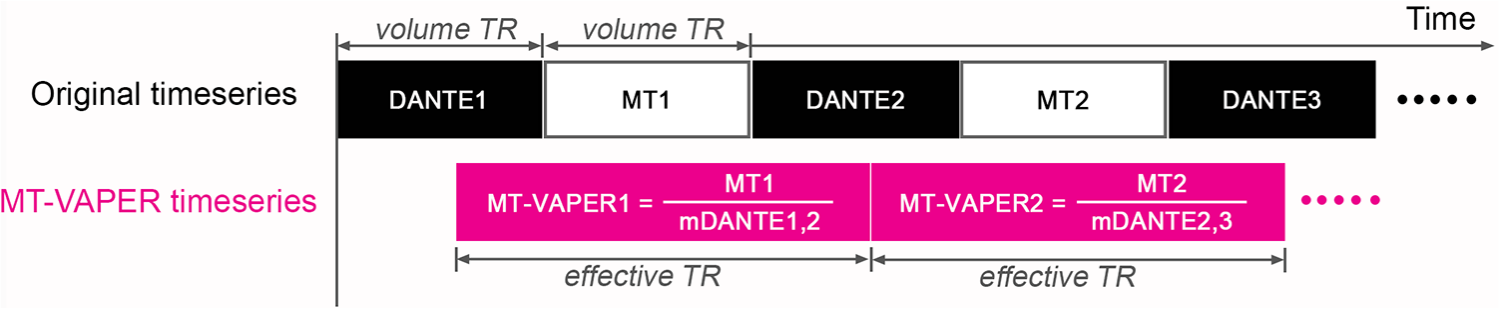
Generation of MT–VAPER time series. Using the VAPER–MT sequence, the original time series alternates between DANTE and MT conditions. Along the time axis, the DANTE1 image is acquired within the 1^st^volume TR and the MT1 image is acquired within the 2^nd^volume TR. The time series of MT–VAPER contrast is generated through dynamic division of neighboring MT and DANTE time points. mDANTE1,2 represents the mean image of DANTE1 and DANTE2. mDANTE2,3 represents the mean image of DANTE2 and DANTE3. The repetition time of MT–VAPER time series is named as the effective TR.

MT–VAPER contrast is generated by dynamically dividing signals from the MT state by those from the DANTE state (Fig. 3). While DANTE and MT images are expected to have the same BOLD$T_{2}^{\text{*}}$weighting, their CBV and CBF weighting differs significantly. Specifically, an increase of CBV leads to a signal decrease in DANTE image but increase in MT image, while an increase of CBF results in a signal increase in MT image. These changes are combined to yield the MT–VAPER contrast, where both CBV and CBF increases produce a unified direction in signal change, as simulated inFigure 4.

**Fig. 4.**
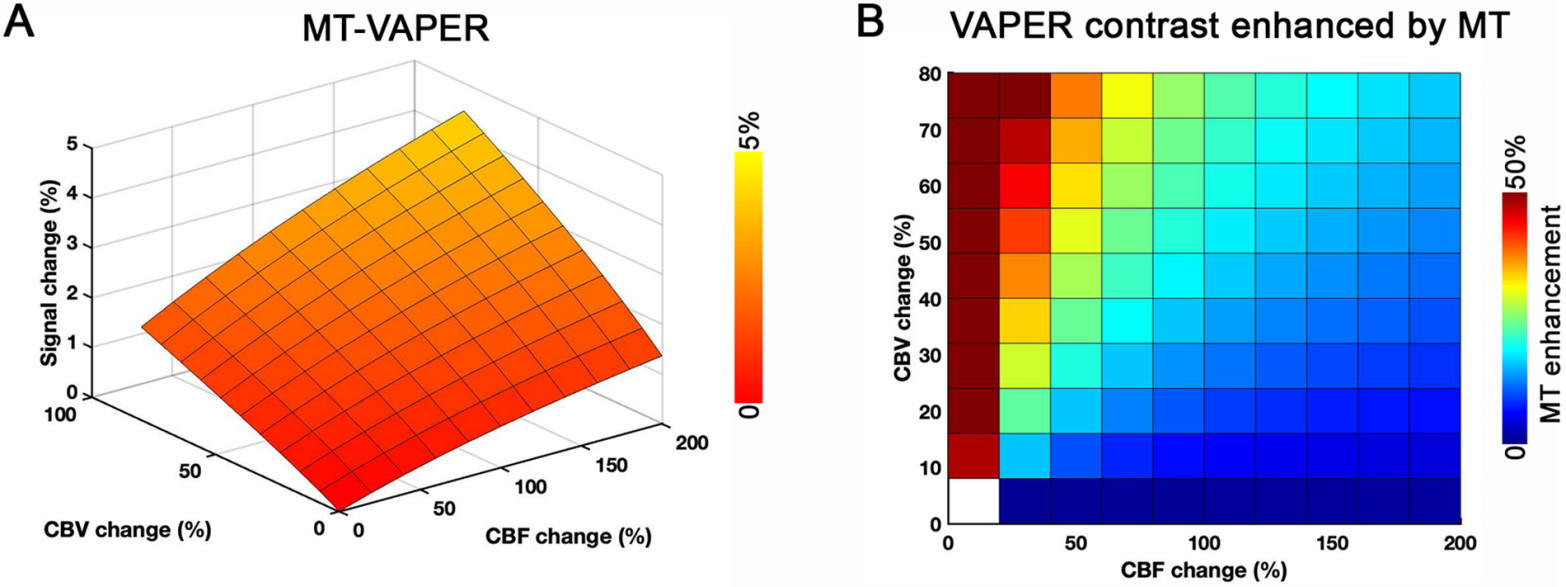
Model simulation of MT–VAPER contrast and its enhancement by MT application. (A) Model simulation demonstrates MT–VAPER’s sensitivity to both CBV and CBF changes. (B) Enhancement of VAPER contrast by MT is shown across a range of CBF and CBV changes. Within typical ranges—a CBF change of 50–100% and a CBV change of 25–60%—MT application boosts the VAPER contrast by 15–35%. This enhancement is more pronounced when CBV changes contribute more than CBF changes to the VAPER contrast, as indicated in the upper left triangular portion of the grid. This pattern suggests that the MT enhancement in VAPER contrast primarily benefits from increased CBV sensitivity.

The signal change detected in the time series of MT state is predominantly contributed by BOLD contrast, thus is named as MT–BOLD in this study.

### 2.5 Model simulation of VAPER acquisition

To theoretically evaluate the signal manipulation within each microvascular and tissue compartment by alternating DANTE and MT acquisitions, the four-compartment model was adopted for Bloch simulations. The specific parameters used for DANTE and MT, as well as the acquisition settings, align with those used in the actual data acquisition. For physiological parameters and relaxation time of the four-compartment model, refer toChai et al. (2020).

Additionally, we simulated how the MT–VAPER signal changes are quantitatively determined by functional CBF and CBV changes.$T_{2}^{*}$change of BOLD contrast was not addressed in the simulation. Functional changes were modeled up to 200% for CBF and 60% for CBV, as these levels of high functional changes have been reported in earlier sub-millimeter fMRI research (T. Jin & Kim, 2008b;Pfeuffer et al., 2002).

## 3 Methods

### 3.1 Image acquisition

All participants gave their informed consent to participate in this study approved by the Institutional Review Board of the University of Illinois at Urbana-Champaign. The experiments were performed on a Terra 7T scanner (Siemens Healthineers, Erlangen, Germany) using a single-channel transmit/32-channel receive head coil (Nova Medical, Wilmington, MA, USA). The VAPER/MT 3D-EPI sequence was implemented to acquire fMRI images of MT–VAPER contrast. DANTE parameters included 100/20 pulses in 1^st^/later segment with pulse interval of 1 ms, pulse duration of 90 µs, flip angle (α) of 9°, and gradient pulse amplitude of 26 mT/m (applied along x and z directions). MT parameters included 36 pulses per segment with 0.2 ms pulse interval, gradients off during preparation, and maximized RF power (10–13°, minimal RF duration allowed under the SAR limit, typically around 90–100 µs). To minimize the direct saturation at the center frequency, we modified the binomial pulse design with a flip angle ramp up at the start and a decreasing ramp at the end of the pulse train, as detailed inSupplementary Figure S1. 3D-EPI acquisition parameters were TE = 18.3 ms, flip angle of water excitation = 18°, 86 slices, 0.8 mm isotropic resolution, 224 × 224 matrix, partial Fourier 7/8 in both phase encoding directions, and CAIPI 3 × 2 (k_z_shift 1) with 1 shot per k_z_-partition (Poser et al., 2013;Stirnberg & Stöcker, 2021). This protocol has an averaging volume TR of 4.27 s, encompassing the preparation and acquisition periods of the entire 3D volume. The effective TR consisting of both DANTE and MT prepared volume acquisitions is doubled as 8.54 s.

In addition, we acquire anatomical MT-weighted EPI images to facilitate data analysis in native fMRI space (Chai, Li, et al., 2021;Chai, Liu, et al., 2021). In the human brain, GM and WM have a significant difference in the fraction of macromolecular hydrogen protons (van Gelderen et al., 2017), thus their contrast can be extracted through MT-weighted imaging and used as anatomical EPI reference. The sequence design is identical to the functional VAPER/MT 3D-EPI imaging. To switch from functional MT–VAPER contrast to anatomical MT weighting, we turned off the DANTE preparation and maximize the RF power of the MT preparation. In this way, we acquired interleaved images between the MT-prepared and control (no preparation) conditions. The MT-weighted anatomical image was generated as$\frac{S_{CTRL}-S_{MT}}{S_{MT}}$, where$S_{CTRL}$represents the image signal in the control condition, and$S_{MT}$represents the image signal of the MT-prepared condition. This combination approach extracts the MT-saturated signal and removes the T_2_* weighting associated with the EPI readout.

### 3.2 Experiment design: Motor finger-tapping task and visual checkerboard stimuli

To demonstrate the laminar specificity of the MT–VAPER contrast for high-resolution fMRI, we conducted experiments involving both motor and visual tasks. Functional MRI data with 86 slices covering both motor and visual cortex were collected to demonstrate the sequence’s feasibility for a large brain coverage.

#### 3.2.1 Motor finger-tapping task

Participants performed a 24-min motor finger-tapping task using the left hand (thumb–index finger), following a paradigm that alternated between rest (25.62 s, corresponding to 6 volume TR’s) and tapping periods (34.16 s, corresponding to 8 volume TR’s). This design targeted a double-layer activity pattern: peak responses in superficial layers reflect inputs from other cortical areas, while peaks in deeper layers represent outputs to the spinal cord, as described inHuber et al. (2017). This distinctive pattern serves as an effective testbed for evaluating the laminar specificity for newly developed layer fMRI methods. In each measurement session, we acquired data of three runs, with each run consisting of eight trials. To assess the reproducibility of layer-dependent activity patterns, three participants underwent duplicate scanning sessions on different days or the same measurement was replicated within a single session.

#### 3.2.2 Visual checkerboard stimuli

The visual task involved a radial black/white checkerboard (size 16° × 10°), with a contrast reversing rate of 10 Hz. The stimulus was presented in a block-design manner, where each active phase lasted for 34.16 s (8 volume TR’s) and alternated with 25.62 s (6 volume TR’s) of rest, across 8 trials per run. Data were collected over three runs per session from four subjects, two of whom participated in two separate sessions, resulting in a total of six sessions.

### 3.3 Comparison with BOLD and the original VAPER without MT enhancement

To evaluate the sensitivity of the MT–VAPER contrast in fMRI, we conducted comparison scans using BOLD and the original VAPER without MT enhancement in six scan sessions. During these comparison sessions, we included additional scans using the previous VAPER 3D-EPI sequence (Chai et al., 2020), by maintaining identical sequence parameters as those used in the VAPER/MT 3D-EPI sequence, with the sole exception that MT preparation was switched off. The same visual checkerboard stimuli were presented during the scan.

### 3.4 Preprocessing and statistical analysis of fMRI data

Motion correction was applied to all functional and anatomical images together within a single session using SPM12 (Wellcome Trust Center for Neuroimaging, London, UK). For the functional data, time series from fixed MT and DANTE images were used to generate the MT–VAPER fMRI contrast time series (Fig. 3). Data points were censored from further analysis whenever the Euclidean norm of the motion derivatives exceeded 0.4 mm or when at least 10% of image voxels were seen as outliers from the trend. For the anatomical data, mean images of fixed control and MT-prepared conditions were used to compute the anatomical reference image (Chai, Li, et al., 2021).

To compute the task-evoked fMRI signal change, a general linear model (GLM) analysis was performed on the time series of each contrast obtained with VAPER/MT 3D-EPI and the original VAPER 3D-EPI sequence, using the AFNI program (Cox, 1996) 3dDeconvolve. To interpret beta weights of each covariate in terms of percentage signal changes, all voxel-wise time series of each contrast were normalized by each voxel’s mean signal across time before incorporating it into the regression model.

### 3.5 Laminar analysis of motor activity induced by finger-tapping task

The ROI used to generate the cortical depth profile (layer ROI) was confined to the lateral side of the hand knob, also known as M1-4a (Terumitsu et al., 2009). The borderlines of CSF/GM and GM/WM in this area were manually drawn on the anatomical MT-weighted EPI image (Fig. 7). To limit inconsistency in M1 ROI definition across sessions for the same subject, we used across-session registration to determine the same ROI location and then manually corrected the boundaries based on the session-specific anatomical EPI image in native fMRI space. For repeated measurements within the same session, an identical M1 ROI was applied. After defining the M1 ROI, we calculated cortical depths based on the equi-volume approach (Waehnert et al., 2014) using the LAYNII software suite (Huber, Poser, et al., 2021) and divided the cortex into 18 equi-volume layers. To calculate activity profiles across layers, all voxels were included in each layer ROI without any statistical thresholding.

The choice to derive 18 layers allowed us to improve layer profile visualization and minimize partial voluming between neighboring voxels (Huber, Poser, et al., 2021;Huber et al., 2017). For a lower number of layers, multiple voxels with centroids across a wider range of cortical depths would have been binned into the same layer, which would have resulted in loss of resolution. In the whole context of this study, we used the term “laminar” or “layer” to indicate a measurement taken along the cortical depth, as opposed to the cytoarchitectonically defined cortical layers.

### 3.6 Laminar analysis of visual response to the checkerboard stimuli

In brain regions such as M1, where spatial atlases are not well defined, the manual selection of ROIs based on anatomical landmarks is a common strategy in layer fMRI analysis (Huber et al., 2017;Persichetti et al., 2020). However, manual ROI selection has potential inconsistencies across participants and studies, which could lead to variations in laminar profiles and complicate replication of findings by other researchers. Such biases in ROI selection can be circumvented in areas like the visual cortex. Here, the availability of a well-fined visual retinotopic atlas allows for the automatic and consistent generation of ROIs across all sessions and participants. Thus, all laminar analysis in the visual cortex can be conducted using an automatic pipeline as below, enhancing the reproducibility and reliability of the results.

First, the MT-weighted EPI images were used to generate WM/GM segments and cortical surface using FreeSurfer (Fischl, 2012) in each individual. Then, based on the automatically generated cortical surface, we calculated cortical depths using the equi-volume approach (Waehnert et al., 2014) with LAYNII software suite (Huber, Poser, et al., 2021) and divided the cortex into 18 equi-volume layers. Next, we used the visual area and eccentricity template from the retinotopy atlas ofBenson et al. (2014)to define the V1 ROI within the stimulus visual field in this study, as V1 with eccentricity less than 10 degree (Benson et al., 2014). Lastly, the visual activity of all voxels without any statistical thresholding within the V1 ROI was extracted and plotted as a function of cortical depth.

## 4 Results

### 4.1 Model simulation

The magnetization evolution of the four-compartment model was simulated as shown inFigure 2Bfor longitudinal magnetization and inFigure 2Cfor transverse magnetization during the readout. The simulations reveal that blood signal is nulled in the DANTE condition while boosted in MT. Specifically, in the DANTE state, blood signal in the arteriolar and venous compartments drops to near-complete suppression. Conversely, during the MT phase, arterioles receive the inflowing fresh blood after the arterial transit time (ATT, which starts at the end of DANTE pulse trains in the preceding DANTE condition), and afterward the magnetization in all microvascular compartments starts increasing. At the same time, tissue signal is suppressed to a lower level by MT compared with that in DANTE state. Transverse magnetization is further weighted by$T_{2}^{*}$and the signal is plotted as a function of EPI excitation with$T_{2}^{*}$decay during EPI readout taken into account. Due to a short$T_{2}^{*}$time, the transverse magnetization of venular blood mostly decays during the EPI readout. Arterioles show the most significant alternation in blood signal, with capillaries exhibiting a lesser extent of change. Tissue signal, in contrast, alternates inversely to the blood signal. It is noteworthy that despite the apparent greater modulation of blood signal compared with tissue, the actual contribution to total voxel signal modulation could be less due to the substantially different volume fractions of blood and tissue in a gray matter voxel (blood of 5.5 mL/100 g, remaining from tissue) (Chai et al., 2020;Lu et al., 2005;Scouten & Constable, 2008).

Figure 4presents the model simulation of MT–VAPER contrast and its enhancement by MT application. The simulation demonstrates that MT–VAPER contrast is sensitive to both CBV and CBF changes. To assess the sensitivity enhancement by MT tissue suppression, we also simulated the VAPER contrast with MT preparation (as used in VAPER–MT 3D-EPI sequence) versus without MT.Figure 4Bshows the enhancement of VAPER contrast by MT across a range of CBF and CBV changes. Within typical ranges—a CBF change of 50–100% and a CBV change of 25–60% during brain activity—MT application boosts the VAPER contrast by 15–35%. This enhancement is more pronounced when CBV changes contribute more than CBF changes to the VAPER contrast, as indicated in the upper left triangular portion of the grid inFigure 4B. This pattern suggests that the MT enhancement in VAPER contrast primarily benefits from increased CBV sensitivity.

### 4.2 Mean images, TSNR, and detection sensitivity of MT–VAPER fMRI

**Fig. 5.**
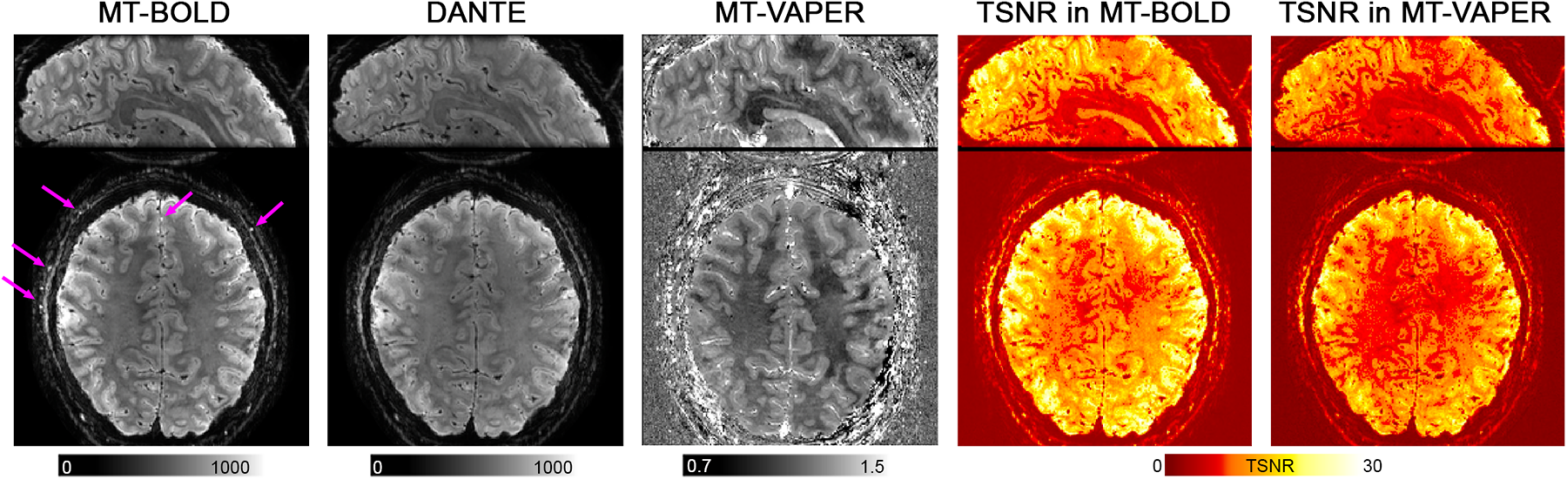
Mean images of MT-prepared and DANTE-prepared volumes from the VAPER–MT 3D-EPI acquisition (24 min, 3 runs) in a representative participant. The purple arrows mark several typical arterial vessels, which are bright in MT image and dark in DANTE. The middle panel shows the derived mean image of MT–VAPER contrast. The right panels display the temporal signal-to-noise ratio (TSNR) maps for both MT–BOLD and MT–VAPER.

Figure 5presents the mean images and temporal signal-to-noise ratio (TSNR) maps of VAPER–MT calculated from 3-runs data (24 min) in one representative participant. The TSNR maps are calculated by dividing the mean by the standard deviation over the time series after detrending. Visible effects of blood suppression appear in arterially dominant voxels, as arterial blood appears bright in MT images yet dark in DANTE images, with several notable examples highlighted by purple arrows. Conversely, venous blood signal is mostly absent due to a rapid$T_{2}^{*}$decay, and microvascular signal is indistinguishable from static tissue signal. GM and WM show differential suppression in MT images, yielding tissue contrast also evident in final MT–VAPER image. A similar result from a single run (8 min) is displayed inSupplementary Figure S2.

To evaluate detection sensitivity, we compared the mean t-value within the visually activated region in V1 using various measurement methods, as shown inFigure 6. And to investigate how much enhancement of the sensitivity can be achieved with MT application, we also compared the sensitivity between the original VAPER and MT–VAPER, where the only difference is the deactivation of MT preparation in the original VAPER. The mean t-values of the original VAPER and MT–VAPER are 35.6% and 46.1% relative to those obtained from BOLD imaging, respectively (two-sided paired t-test, p < 0.001 for both BOLD vs. original VAPER and BOLD vs. MT–VAPER). Notably, MT application resulted in a 29.5% increase in VAPER sensitivity over the non-MT version (two-sided paired t-test, p < 0.001 for original VAPER vs. MT–VAPER), aligning closely with the predicted enhancement from the stimulation (15–35%).

**Fig. 6.**
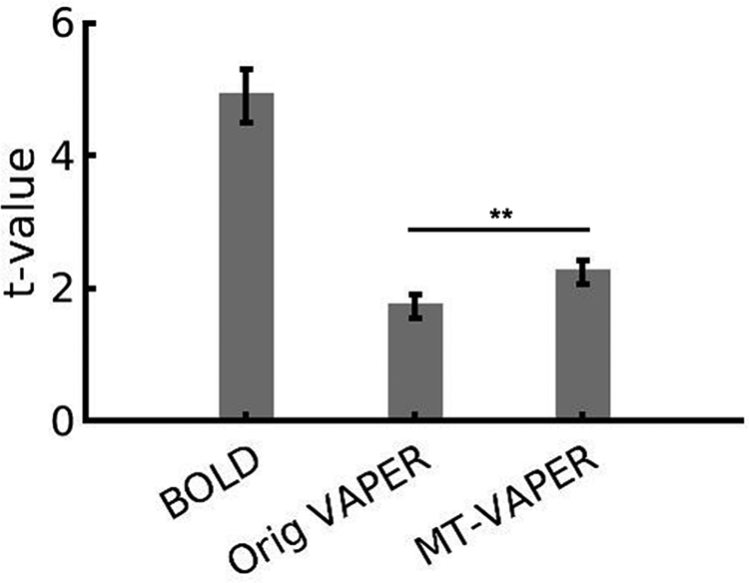
Comparison of detection sensitivity of BOLD (no preparation, normal 3D-EPI), original VAPER (DANTE on but MT off), and MT–VAPER (both DANTE and MT on). It shows the mean t-values in the BOLD activated region by visual stimulus within V1 for different measurement methods. The mean t-value of original VAPER and MT–VAPER is 35.6% and 46.1% of that from BOLD, respectively (two-sided paired t-test, p < 0.001 for both BOLD vs. original VAPER and BOLD vs. MT–VAPER). Notably, MT application results in a 29.5% increase in VAPER sensitivity over the original VAPER without MT (** for p < 0.001, original VAPER vs. MT–VAPER). Error bars represent ±SEM across six sessions.

### 4.3 Laminar specificity of MT–VAPER contrast

In order to demonstrate the laminar specificity of MT–VAPER fMRI, we analyzed the laminar profile of MT–VAPER signal changes induced by motor finger-tapping and visual checkerboard stimuli in each corresponding cortical area, respectively.

**Fig. 7.**
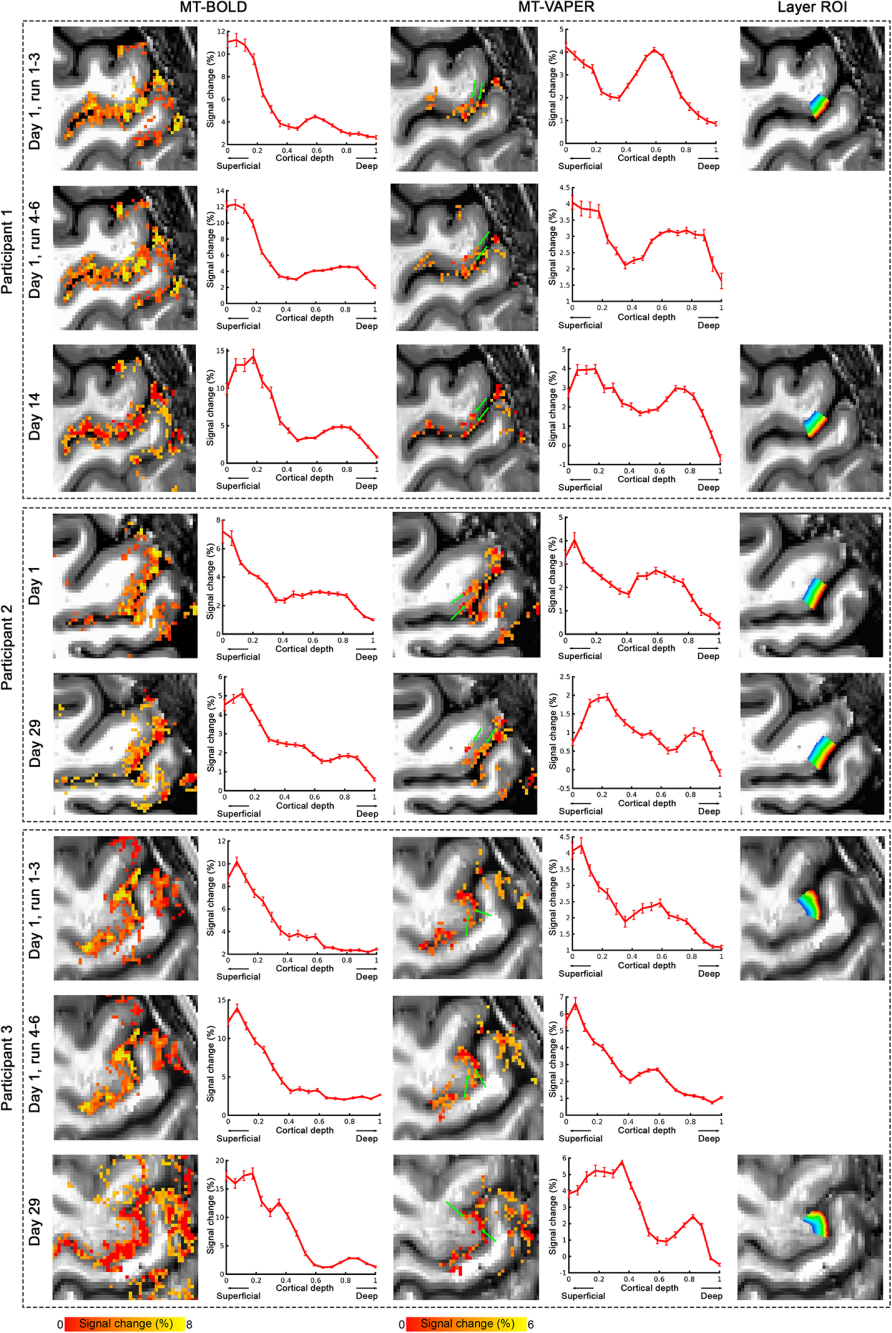
Layer-dependent fMRI response in the motor cortex across participants and sessions. Each column represents a different fMRI contrast, while each row corresponds to scans from different participants. For each scan, activity maps of a square area centered on M1-4a are shown for both MT–BOLD and MT–VAPER contrasts. The underlays are the anatomical EPI images from an identical acquisition with functional imaging. The percentage signal changes within layer ROIs are plotted across different cortical depths for each contrast.

Figure 7shows the layer-dependent response to motor finger-tapping task, measured by both MT–BOLD (only from MT-prepared EPI volumes) and MT–VAPER (dynamical division of MT by DANTE time series) contrasts from different participants and in different measurement sessions/runs of each participant. The activation maps were thresholded at p < 0.05 with applying cluster size >40. Activity maps are overlaid on the mean images of anatomical EPI and the signal changes across cortical depth in layer ROIs of M1-4a area are shown to the right side, accordingly. In MT–VAPER activation maps, the activity in superficial and deep layers is distinguishable in the position marked by green arrows. In the corresponding layer-dependent profiles of brain activity, MT–VAPER contrasts clearly show two response peaks in different laminae. The peak responses in superficial and deep layers are suggested to reflect an input signal from other cortical areas and an output signal to the spinal cord, respectively (Huber et al., 2017). This double peak response feature is highly reproducible across individuals and across different measurement repetitions (upper and bottom row of each subpanel inFig. 7). In the MT–BOLD activity map, the task-evoked response shows a strong increase toward cortical surface, and the distinctions between layers are much less apparent. Because the signal change of MT–BOLD measurement is mostly contributed by BOLD contrast as opposed to CBF or CBV, the laminar feature of the MT–BOLD activity map is largely determined by the specificity of BOLD contrast. The group-mean laminar profiles for BOLD and MT–VAPER are presented inFigure 8, with normalized profiles shown inSupplementary Figure S3.

**Fig. 8.**
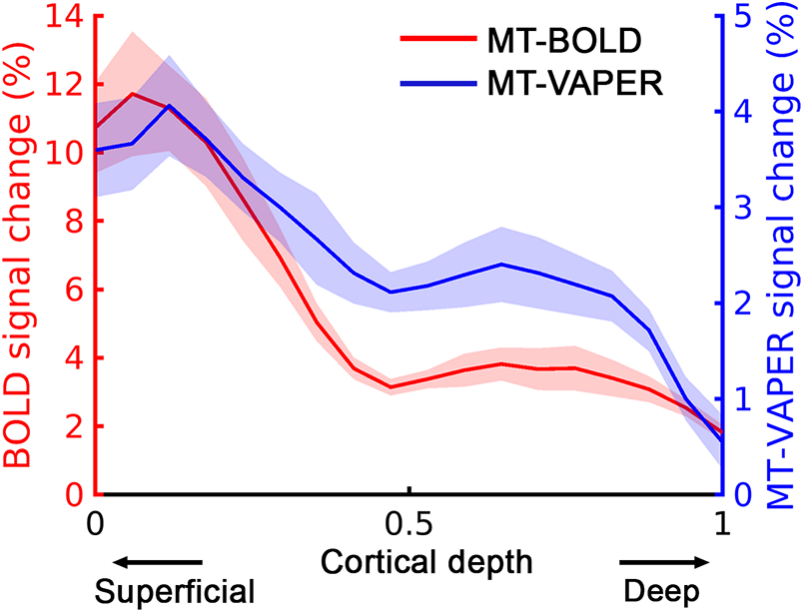
Group mean laminar profiles of fMRI response in the motor cortex. MT–BOLD responses are depicted with a red curve, and MT–VAPER responses with a blue curve. Shaded areas represent ± SEM across different measurements fromFigure 7.

**Fig. 9.**
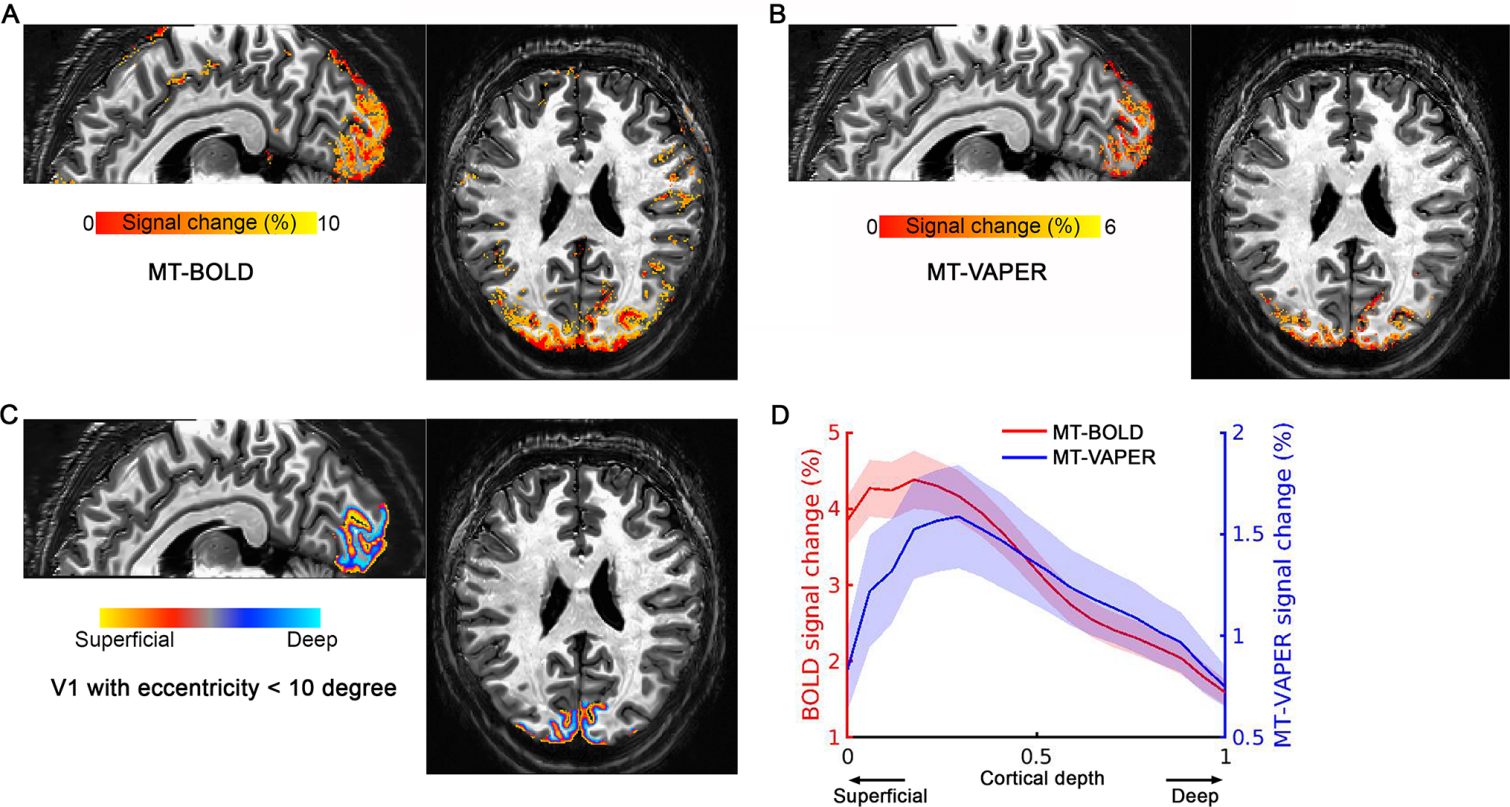
Activation maps and the laminar profiles of MT–BOLD and MT–VAPER response to the visual checkerboard stimuli. (A) MT–BOLD activation map. (B) MT–VAPER activation map. (C) Cortical depth within V1 with eccentricity less than 10 degree (within visual field). The underlay is the anatomical EPI image from an identical acquisition with functional imaging. (D) MT–BOLD and MT–VAPER signal changes as a function of cortical depth. Shaded areas represent ± SEM across sessions.

Figure 9shows the laminar response to visual checkerboard stimuli, measured by MT–BOLD and MT–VAPER within the visual stimulus field in V1. The activity maps from one representative participant are shown inFigure 9Afrom MT–BOLD measurement andFigure 9Bfrom MT–VAPER contrast.Figure 9Cshows the cortical depth reconstructed within V1 with eccentricity less than 10 degrees, which is the visual field size of the visual stimuli.Figure 9Dshows the corresponding laminar profiles of MT–BOLD and MT–VAPER activity during visual checkerboard stimulation, averaged over six sessions. The BOLD response is biased toward the cortical surface due to the draining vein effect, with signal change decreasing from the cortical surface near CSF (depth of 0) to the deeper cortical layers near WM (depth of 1), as shown in red curve with a peak at depth 0.116 based on Gaussian peak fitting (O’Haver, 1997). In contrast, MT–VAPER response (blue curve) exhibits less superficial bias and peaks closer to the middle cortical depths (peak at depth 0.411). This laminar feature is consistently seen across individuals as shown inFigure 10, with MT–BOLD laminar profiles peaking at 0.125 ± 0.070 and MT–VAPER profiles peaking at 0.410 ± 0.038 (mean ± standard deviations across individuals). A further comparison of the laminar profiles for GE–BOLD, MT–BOLD, original VAPER, and MT–VAPER is presented inSupplementary Figure S4. Both BOLD measurements exhibit a similar superficial laminar bias. The laminar profiles of the original VAPER and MT–VAPER are also similar, with peaks at depths of 0.38 and 0.41, respectively. This consistency in laminar specificity is expected as both VAPER contrasts share the same CBV and CBF-based origins.

**Fig. 10.**
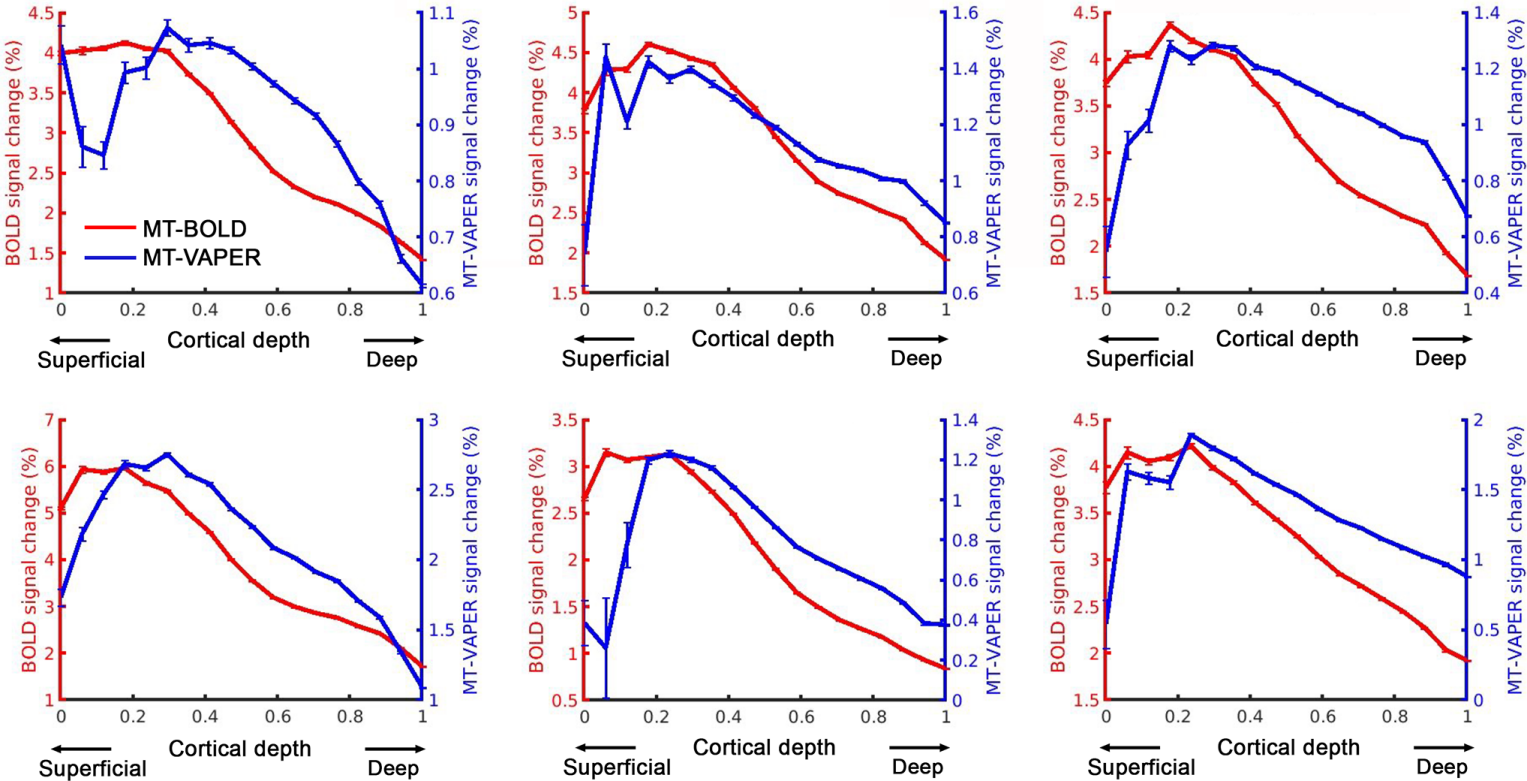
Individual results of BOLD and MT–VAPER signal changes as a function of cortical depth in V1 with eccentricity less than 10 degree. Error bars represent ± SEM across voxels within ROI.

## 5 Discussion

In this study, we introduced a novel sequence tool for laminar fMRI research, designed to acquire MT-enhanced VAPER contrast. Compared with the original VAPER, incorporating MT yields approximately a 30% improvement in VAPER sensitivity. At the same time, the laminar specificity remains superior to both GE–BOLD and MT–BOLD, showing a significantly reduced superficial bias. Thus, MT–VAPER emerges as another valuable addition among a variety of non-BOLD methods available for laminar fMRI. Below, we discuss the underlying mechanisms by which MT enhances VAPER relative to the original version, consider how these findings relate to other MT-related approaches in the literature, and examine potential limitations and considerations for MT–VAPER as a new non-BOLD laminar fMRI modality.

### 5.1 MT application in VAPER enhances CBV contrast and maintains superior laminar specificity

In this study, we employed MT to introduce a tissue suppression-based VASO effect (the inverse of blood-nulling VASO), which adds additional CBV sensitivity to the original VAPER contrast. Traditionally, MT has been utilized to differentially suppress the signal from gray matter relative to blood. The blood signal from capillary and venous compartment can also be affected by MT due to the exchange of MT-suppressed water signal from tissue, and those exchanged spins flow downstream to the venous blood pool. In contrast, the arterial and arteriolar blood pools experience only minimal MT effects due to the inflow of fresh blood spins that are unaffected by MT. Assuming a free water exchange between capillary and tissue, MT-insensitive arterial blood signals can be separated from MT-dependent extravascular tissue and venous blood, and this approach has been used to measure arterial-specific CBV changes in fMRI (T. Kim & Kim, 2010,^2011^). However, in fact, the water exchange between capillary and tissue is limited by a permeability parameter as modeled in this study. Nonetheless, the CBV change detectable by MT is still dominated from the arterial and arteriolar compartment by noting that (1) the contribution of venous blood to the overall CBV change is minimal due to the very short T2* of venous blood at 7T (Chai et al., 2020) and (2) the predominant increase in total CBV during neural activation originates from arterial and arteriolar rather than venous blood volume changes (T. Kim et al., 2007). Consequently, the MT application in this study enhances the same arterial/arteriolar CBV component targeted by the original VAPER method. Hence, MT–VAPER not only boosts sensitivity through CBV but also maintains similar laminar specificity as the original VAPER, given their shared vascular origins.

### 5.2 Sensitivity of VAPER, MT–VAPER, and standalone MT for fMRI

Incorporating MT into the VAPER sequence aims to address the sensitivity limitations inherent in CBV and CBF-based fMRI techniques compared with traditional BOLD imaging. By integrating a long stream of small flip angle binomial MT pulses, we achieved significant enhancements in VAPER sensitivity. There are two intravascular signal components in the original VAPER. During the DANTE prepared image acquisition, the blood signal within the microvasculature of the human brain is nearly nulled to achieve a VASO contrast. Following this post-DANTE, fresh blood from regions beyond the coil coverage flows into the imaging area, replacing the nulled blood and generating a perfusion-weighted contrast. In this study, we add MT pulses at the same time when fresh blood flows into the brain, thus the tissue signal is suppressed, serving as an inverse VASO, adding additional CBV sensitivity to the original VAPER method. Our simulations predicted an 15–35% enhancement in VAPER signal changes with MT application, and empirical data showed a 29.5% increase in sensitivity compared with the original VAPER method without MT. In practice, this enhancement will depend on the actual MT pulse power and its suppression effect allowed by the scanner SAR limit.

Despite the considerable enhancements MT brings to VAPER sensitivity, it is unlikely that MT alone could serve as an independent contrast method for fMRI. This study demonstrated that while MT enhances VAPER sensitivity significantly, MT alone achieves only about 30% of the sensitivity offered by the original VAPER. Given that the original VAPER’s sensitivity is already less than half as sensitive as BOLD imaging (Chai et al., 2020), using MT alone for fMRI is impractical due to its low sensitivity. This underscores the necessity to combine it with other contrast mechanisms, such as MT–VAPER in this study and arterial blood contrast (ABC) approaches previously explored (Pfaffenrot & Koopmans, 2022;Priovoulos et al., 2023;Schulz et al., 2020).

### 5.3 Comparison with other MT applications for fMRI in the literature

While our primary goal was to employ MT to enhance CBV sensitivity in VAPER, other studies have explored MT differently. Some have incorporated MT into BOLD-based measurements and investigated strategies to improve its specificity.

Previous animal research has extensively examined the effects of MT on BOLD-fMRI specificity. In conventional GE–BOLD fMRI without MT, the highest signal changes occurred at the cortical surface, where large draining veins are located. However, as the MT level increases, percentage signal changes in the intracortical regions increase, while those at the cortical surface remain unchanged (T. Kim & Kim, 2010). This indicates that MT–BOLD does improve the intracortical specificity, but it still exhibits the same superficial bias observed in conventional BOLD. These observations align with our findings in this study. In our control measurements of MT-prepared EPI (referred to as MT–BOLD), the superficial bias from BOLD remains dominant, as evident inFigures 7–10. Comparing GE–BOLD and MT–BOLD inSupplementary Figure S4shows that while MT–BOLD slightly improves intracortical specificity (indicated by a subtle inward shift of the laminar profile), the superficial laminar bias is largely similar for both BOLD methods.

To further mitigate superficial bias in MT–BOLD, strategy like reducing TE has been explored in recent research (Pfaffenrot & Koopmans, 2022;Priovoulos et al., 2023;Schulz et al., 2020). By reducing TE, the relative contribution of CBV from MT is increased in the total MT–BOLD signal, while the superficial bias from BOLD signal can be reduced. Alternatively, this BOLD-related superficial laminar bias can be corrected by simply comparing the signal changes of MT–BOLD with the original BOLD without MT. This approach has been shown to achieve superior laminar specificity without superficial bias (T. Kim & Kim, 2010). This strategy is similar as we applied in MT–VAPER sequence design through an interleaved acquisition. However, the sensitivity of detecting standalone MT-related signal changes after removing the BOLD contribution is lower than other CBV or CBF-based fMRI measurements such as VASO, VAPER, and MT–VAPER, primarily due to the SAR limit at high-field strengths in human fMRI.

### 5.4 Practical considerations and potential limitations of MT–VAPER

#### 5.4.1 Long repetition time

One of MT–VAPER’s main limitations is its long repetition time. Unlike BOLD-fMRI, where the minimum repetition time is primarily constrained by gradient performance and achievable acceleration with parallel imaging, non-BOLD fMRI requires additional acquisition time due to slower intravascular contrast generation and interleaved acquisitions for BOLD correction. This challenge is common to VAPER (Chai et al., 2020), ASL (Shao et al., 2024), and VASO (Huber, Ivanov, et al., 2018;Huber, Tse, et al., 2018;Huber et al., 2014) techniques. Compared with BOLD-fMRI, MT–VAPER requires additional interleaved acquisition and preparation time, offering dual contrast measurements of both MT–VAPER and BOLD, but at the cost of effective TR that is 2.3 times that of the original 3D-EPI. The extended TR in MT–VAPER not only reduces temporal efficiency but also complicates BOLD correction. The effectiveness of BOLD correction relies on the assumption that BOLD signal is not changing significantly between consecutive time points. However, during a long TR period, significant variations in BOLD signal can occur, resulting in a sub-optimal BOLD correction with a certain level of BOLD contribution in MT–VAPER time series.

A promising method to expedite VAPER acquisition in the future is the application of time-dependent low-rank acceleration and reconstruction techniques (R. Jin et al., 2023,^2024^;Liang, 2007). Unlike conventional time-independent parallel imaging methods, the low-rank approach leverages the temporal information during image reconstruction, providing a greater degree of acceleration than time-independent methods.

#### 5.4.2 B1+/B0 field inhomogeneity and SAR

MT–VAPER is sensitive to the transmit amplitude B1+ field inhomogeneity, particularly when the RF power of MT binomial pulses is insufficient. At ultra-high fields, the B1+ field can be quite inhomogeneous over the brain. A higher RF power of MT pulses can increase tissue suppression, enhancing CBV and the overall MT–VAPER sensitivity. This study utilized the highest RF power allowed by SAR limits to optimize MT preparation. However, any B1+ field inhomogeneity could result in spatial variance in MT effect, affecting the overall MT–VAPER sensitivity. In contrast, B1+ field inhomogeneity does not critically impact DANTE preparations as long as its flip angle is not dramatically reduced (e.g., remains above 50% of intended) (Chai et al., 2020). Variation in flip angle mainly influences the time or number of pulses needed (200–400) to achieve the blood signal saturation state. With a total of 1780 DANTE pulses per volume TR utilized in this study, the blood signal remains nulled throughout most of the DANTE state despite variations in flip angle. To mitigate the effects of B1+ inhomogeneity on MT and prevent significant degradation of DANTE flip angle, employing a parallel transmit system with B1 shimming is an effective solution.

As for B0 inhomogeneities, MT–VAPER is not expected to be more susceptible than the original 3D-EPI. Both DANTE and MT pulses are not sensitive to the B0 inhomogeneities, as the frequency shifts caused by B0 inhomogeneity is very small compared with the saturation band of MT binomial pulses (±2500 Hz off-resonance frequency as shown inSupplementary Fig. S1) and DANTE is theoretically insensitive to B0 inhomogeneity (Li et al., 2012). EPI artifacts associated with B0 inhomogeneities are expected to be consistent across DANTE-prepared and MT-prepared EPI images, thus are likely to be canceled out when computing their ratio.

#### 5.4.3 Head-only transmit coil requirement

One necessary condition for generating perfusion weighting into the MT–VAPER contrast is that the blood outside the head or imaging region remains unaffected by DANTE suppression, allowing it to flow into the imaging volume to replace the nulled blood during MT state. This generates a perfusion-weighted signal while MT simultaneously suppresses tissue signal. Therefore, a head-only transmit coil is essential to prevent blood outside the coil coverage from being nulled during DANTE preparation. The arterial transit time (ATT) is a critical factor, reflecting the duration it takes for fresh blood from outside the coil coverage to reach the voxel microvasculature. The ATT can vary significantly depending on the distance from outside the coil coverage to the image regions, which differs across brain areas and participants (Mildner et al., 2014;Qiu et al., 2010), as well as the coil profile, which reflects how the transmit amplitude B1+ falls off outside the coverage. These variations introduce uncertainty into the perfusion weighting of MT–VAPER contrast. In this study, the interval from the end of DANTE blood nulling to the k-space center of the MT acquisition is about 2 s, which is sufficient for fresh blood to reach the imaging regions’ microvasculature.

#### 5.4.4 Misleading about using TSNR to assess MT–VAPER sensitivity

While TSNR is commonly used to evaluate sensitivity in BOLD fMRI, it becomes misleading for assessing MT–VAPER sensitivity. When applying MT with VAPER, we aim for a substantial tissue signal suppression in MT-prepared EPI to enhance VAPER sensitivity to CBV changes. This suppression, while lowering the MT–VAPER TSNR due to reduced signal intensity, does not imply reduced sensitivity. In fact, this tissue signal suppression could reduce MT-prepared EPI’s sensitivity to BOLD signal changes but increase its sensitivity to CBV changes. And in MT–VAPER, we integrate CBV and CBF weighting while factor out BOLD contribution. Hence, stronger MT-induced tissue suppression leads to more MT-enhanced VAPER sensitivity—a relationship that TSNR fails to reflect and even suggests the opposite. For instance, if there is no MT suppression, MT–VAPER reverts to the original VAPER. This condition would lead to a reduced VAPER sensitivity as there is no additional CBV sensitivity generated by MT effect, however, the TSNR shows the opposite as the signal intensity is higher with no tissue signal suppression. Therefore, using TSNR to optimize MT–VAPER and evaluate its sensitivity is misleading and incorrect. Instead, sensitivity in this study is assessed through the statistical power of t-values for the activity detection.

## 6 Conclusions

In this study, we developed an innovative MT-enhanced VAPER imaging sequence that enhances sensitivity by 30% compared with traditional VAPER without MT, while maintaining superior laminar specificity without increasing acquisition time. By integrating a magnetization transfer approach to induce an inverse VASO effect within the existing VAPER framework, this method alternates between DANTE blood-nulling and MT-tissue-suppression states to create a combined contrast that boosts overall sensitivity. Utilizing a long stream of small flip angle binomial pulses for MT, this technique is compatible with variable EPI acquisition segmentations and complies with SAR requirements at 7T human scanners. Empirically validated in human primary motor and visual cortices, MT–VAPER demonstrates superior laminar specificity and reproducibility, highlighting its potential as a valuable non-BOLD alternative for layer-specific fMRI studies in human brain research.

## Supplementary Material

Supplementary Material

## Data Availability

Data analysis uses publicly available software packages, including FreeSurfer (https://freesurfer.net/), AFNI+SUMA (https://afni.nimh.nih.gov/), SPM (https://www.fil.ion.ucl.ac.uk/spm/), ANTs (http://stnava.github.io/ANTs/), and LAYNII (https://github.com/layerfMRI/LAYNII). The datasets can be made available from the corresponding author upon request.

## Appendix

Mathematical modeling of magnetization dynamics

### A.1. Four-compartment model

This appendix outlines the temporal characteristics of longitudinal magnetization for a general four-compartment model encompassing tissue, capillary, arteriole, and venule compartments, with considerations for relaxation, blood flow, and volume, and permeability (PS):

- **Tissue compartment:**
  $$\frac{\text{d}{\text{M}}_{\text{z},\;\text{tissue}}\left(\text{t}\right)}{\text{dt}}=\frac{M_{0,\;tissue}-M_{z,\;tissue}\left(t\right)}{T_{1,\;tissue}}+\;\frac{PS}{\left(100-CBV_{total}\right)\;\cdot \;\rho_{tissue}}\;\cdot \;(M_{z,\;capillary}\left(t\right)-M_{z,\;tissue}\left(t\right)).$$
- **Capillary compartment:**
  dMz, capillary(t)dt=M0, capillary−Mz, capillary(t)T1, capillary+ CBFCBVcapillary · ρblood · (Mz, arteriole(t)−Mz, capillary(t))+ PSCBVcapillary · ρblood · (Mz, tissue(t)−Mz, capillary(t)).
- **Arteriole compartment:**
  $$\frac{\text{d}{\text{M}}_{\text{z},\;\text{arteriole}}\left(\text{t}\right)}{\text{dt}}=\frac{M_{0,\;arteriole}-M_{z,\;arteriole}\left(t\right)}{T_{1,\;arteriole}}+\;\frac{CBF}{CBV_{arteriole}\;\cdot \;\rho_{blood}}\;\cdot \;\left(M_{z,\;inflow}\left(t\right)-M_{z,\;arteriole}\left(t\right)\right).$$
- **Venule compartment:**
  $$\frac{\text{d}{\text{M}}_{\text{z},\;\text{venule}}\left(\text{t}\right)}{\text{dt}}=\frac{M_{0,\;venule}-M_{z,\;venule}\left(t\right)}{T_{1,\;venule}}+\;\frac{CBF}{CBV_{venule}\;\cdot \;\rho_{blood}}\;\cdot \;\left(M_{z,\;capillary}\left(t\right)-M_{z,\;venule}\left(t\right)\right),$$

where$M_{z,\;inflow}\left(t\right)$is manipulated by DANTE pulse trains in the same way as in arterioles and set to 1 after fresh blood arriving in the arteriole after the DANTE condition. The transversal plane magnetization can be similarly described, substituting$T_{1}$relaxation terms with$T_{2}$relaxation terms. Inflowing spin always has zero magnetization in the transversal plane, assuming it is not affected by readout. The further equations to describe the DANTE effect have already been explained inLi et al. (2012)and are not listed here. All physiological parameters and relaxation times that are used in the model have been listed inChai et al. (2020).

### A.2. Two-pool Bloch equations for MT effect modeling

The longitudinal magnetization dynamics for gray matter tissue, under the influence of MT, are governed by two-pool Bloch equations:

- **Macromolecular Protons (MP):**
  $$\frac{dM_{z,\;MP}\left(t\right)}{dt}=\;\left(f-M_{z,\;MP}\left(t\right)\right)R_{1,\;MP}-RM_{z,\;MP}\left(t\right)-\frac{k}{f}M_{z,\;MP}\left(t\right)+\frac{k}{1-f}M_{z,\;WP}\left(t\right).$$
- **Water Protons (WP):**
  $$\frac{dM_{z,\;WP}\left(t\right)}{dt}=\;\left(1-f-M_{z,\;WP}\left(t\right)\right)R_{1,\;WP}-2\pi B_{1}M_{y,\;WP}\left(t\right)-\frac{k}{1-f}M_{z,\;WP}\left(t\right)+\frac{k}{f}M_{z,\;MP}\left(t\right).$$

Assuming$B_{1}$is applied along x direction; R is the saturation rate and is proportional to$B_{1}^{\;2}$(Portnoy & Stanisz, 2007) (we assume a 2% saturation with a 12°, 100 us hard pulse based on the calibration inChai, Li, et al. (2021), R for other pulse parameter scales with$B_{1}^{\;2}$relative to this reference);fis fraction of macromolecular protons, assumed to be 10% in gray matter;kis the exchange rate, assumed to be 1.45 s^-1^;$R_{1,\;WP}$and$R_{1,\;MP}$are assumed to be 2.0 s^-1^and 0.4 s^-1^, respectively. More theoretical background about this model can be found inWang et al. (2020). MT is assuming no direct effect on vascular compartments, but will impact them through permeability exchange and downstream flow as described in the four-compartment model.
