# Supplementary material for: Blood nulling versus tissue suppression: Enhancing integrated VASO and perfusion (VAPER) contrast for laminar fMRI
Source: Imaging Neurosci (Camb). 2025 Jan 21;3:imag_a_00453. doi: 10.1162/imag_a_00453 (PMC12319795; doi:10.1162/imag_a_00453)
Supplement: Supplementary Material [file imag_a_00453-supp.pdf]

SUPPLEMENTARY MATERIALS

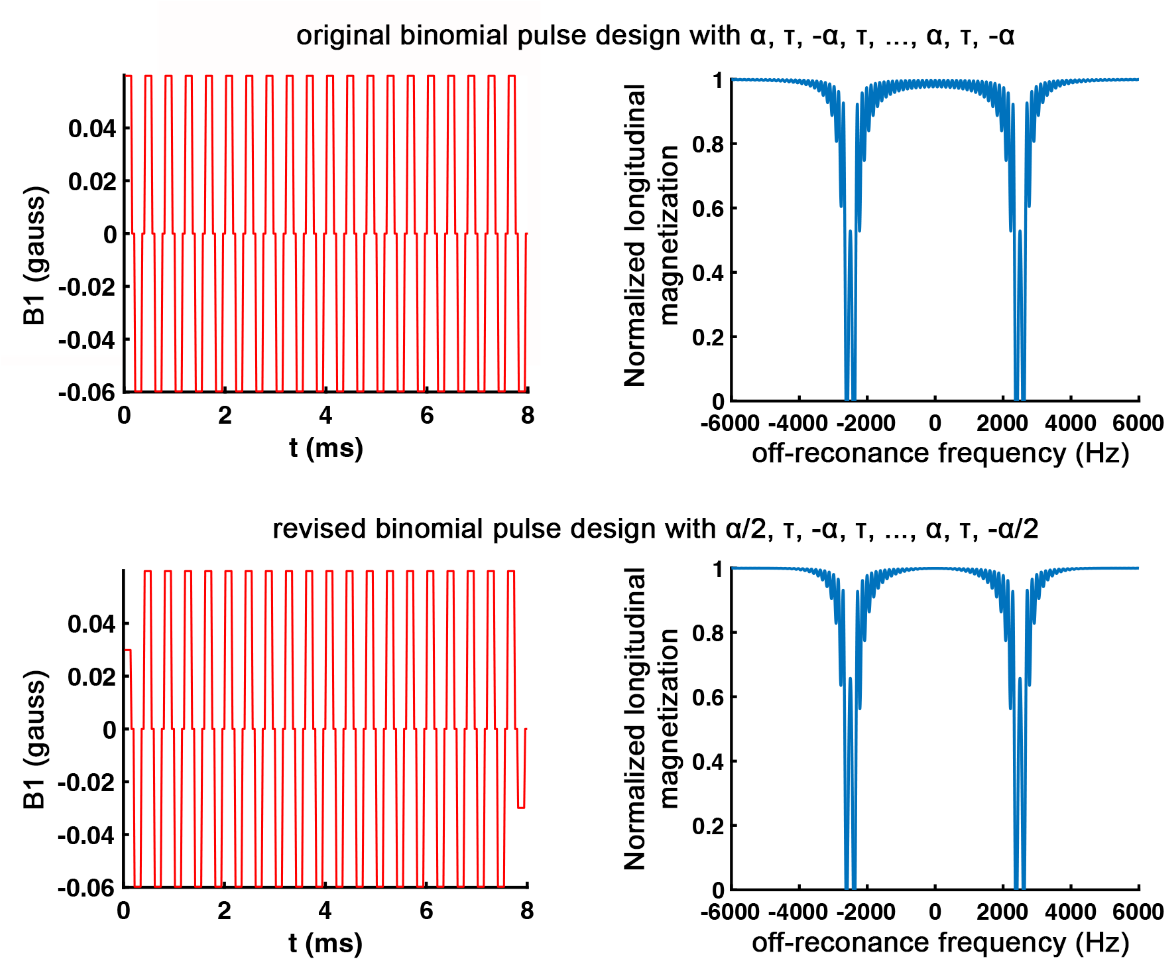

Figure S1. Top row depicts the original binominal pulse design with constant flip angle (α) and pulse interval (τ), as α, τ, -α, τ, ..., α, τ, -α. Bottom row displays the revised binominal pulse design incorporating a flip angle ramp at the beginning and at the end of the pulse train, specifically α/2, τ, -α, τ, ..., α, τ, -α/2. The left column of each row plots of B1 amplitude of the binomial pulse trains over time (40 pulses total, 0.2 ms pulse interval); the right column presents frequency spectra computed via Bloch simulation. The revised binomial pulse design exhibits noticeably less saturation at the center frequency compared to the original and is used in our MT-VAPER sequence.

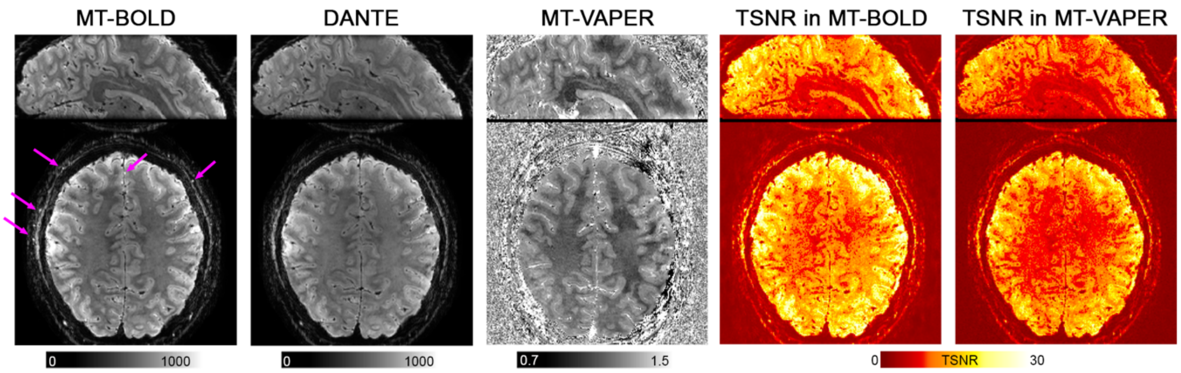

Figure S2. Mean images of MT-prepared and DANTE-prepared volumes from the VAPER-MT 3D-EPI acquisition in one run (8min) from a representative participant. The purple arrows mark several typical arterial vessels, which are bright in MT image and dark in DANTE. The middle panel show the derived mean image of MT-VAPER contrast. The right panels display the temporal signal-to-noise ratio (TSNR) maps for both MT-BOLD and MT-VAPER.

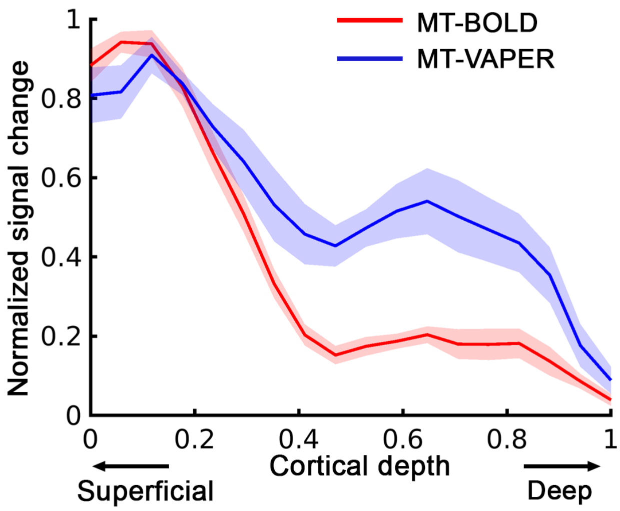

Figure S3. Group mean laminar profiles of fMRI responses in the motor cortex. Signal changes are normalized to a 0–1 range for better comparison of laminar profiles. Shaded areas represent ±SEM across different measurements.

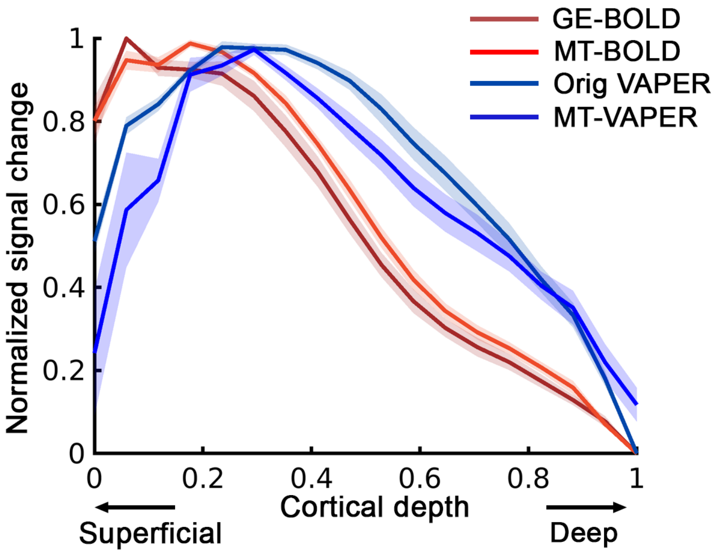

Figure S4. Comparison of laminar profiles of visual response measured using different contrast methods. Signal changes are normalized to the 0-1 range and then averaged across individuals, with shaded areas representing ±SEM. Gaussian peak fitting identifies the profile peak at cortical depth as follows: 0.03 for GE-BOLD, 0.12 for MT-BOLD, 0.38 for original VAPER and 0.41 for MT-VAPER.
